# The Effect of Different Organic Acids and Their Combination on the Cell Barrier and Biofilm of *Escherichia coli*

**DOI:** 10.3390/foods12163011

**Published:** 2023-08-10

**Authors:** Qing-Yang Ji, Wenqiong Wang, Haodong Yan, Hengxian Qu, Yang Liu, Yi Qian, Ruixia Gu

**Affiliations:** College of Food Science and Engineering, Yangzhou University, Yangzhou 225127, China; jiqingyang9@gmail.com (Q.-Y.J.); wenqiong.happy@163.com (W.W.); 15733201233@163.com (H.Y.); quhengxian@163.com (H.Q.); lyhsm1996@163.com (Y.L.); qy824632710@163.com (Y.Q.)

**Keywords:** organic acid, *Escherichia coli*, synergistic bacterial inhibition, cell barrier, biofilm

## Abstract

Organic acids are natural antimicrobial compounds commonly used in the food industry. In this study, acetic, lactic, butyric, citric, and malic acid at minimum inhibitory concentrations and their combinations at optimal inhibition concentrations were used to treat *E. coli*, and the effects on the cell barrier and biofilm of *E. coli* were evaluated. Acetic acid showed the highest membrane-damaging effect, while citric acid and malic acid could specifically damage the cell wall of *E. coli*, leading to alkaline phosphatase leakage. The RT-qPCR results showed that organic acids upregulated the membrane-protein-related genes of *E. coli*, and the combination of organic acids had a wider range of effects than single organic acid treatment. Moreover, organic acids inhibited the formation of *E. coli* biofilm and cellular activity within the biofilm. This study showed that the combination of organic acids plays a synergistic inhibitory role mainly through multiple destructive effects on the cell barrier and exhibited synergistic anti-biofilm effects. The three–three combination of acetic, lactic acid, and a third organic acid (butyric, citric, or malic) can play a better synergistic antibacterial effect than the two-pair combination of acetic and lactic acid. These findings have implications for the usage, development, and optimization of organic acid combinations.

## 1. Introduction

Foodborne disease outbreaks are currently a major global food safety concern. The World Health Organization estimates that approximately 600 million people become ill each year after consuming contaminated food, resulting in 420,000 deaths and the loss of 33 million healthy life years [1]. One of the main pathogens causing these foodborne cases is *E. coli*. It can cause a variety of serious diseases such as hemorrhagic proctitis, hemolytic uremic syndrome, and acute renal failure [2]. Biofilms are microbial cell aggregates in extracellular polymeric substances (EPSs). In the food industry, most foodborne pathogens rely on biofilms to adhere to food contact surfaces [3]. *E. coli* can easily form biofilms on the surface of living and non-living carriers, leading to the cross-contamination of food. Therefore, the control of *E. coli* and its biofilm contamination is extremely important in the food industry.

Inorganic substances such as sodium hypochlorite and quaternary ammonium compounds are commonly used as disinfectants in the food industry to inactivate pathogenic bacteria in food and on food-processing surfaces [4,5]. However, due to the potential toxicity of disinfectants and their by-products, as well as the increase in the tolerance of pathogenic bacteria to such disinfectants [6,7,8], it is necessary to develop alternative strategies for inorganic disinfectants to improve the microbial safety of food without producing toxic by-products. Bacteriocins are low molecular weight antimicrobial peptides synthesized by bacterial protein synthesis apparatus and they inhibit other bacteria [9]. Antimicrobial peptides are small-molecule proteins with bacteriostatic activity that are widely available in living organisms, can be used for food preservation, and are harmless [10]. However, the problems of bacteriocins and antimicrobial peptides in terms of their bacteriostatic activity, industrial productivity, and purification efficiency have limited the application of these two substances in the food industry; nisin is the only bacteriocin currently approved by the FDA for use as a food preservative [11]. Lactoferrin, lactoperoxidase, herbs, spices, and chitosan are also natural antimicrobial compounds that can be used in foods [12], but their material properties limit their large-scale application in the food industry.

Organic acids are natural compounds found in various foods and are mainly produced by some microorganisms. Organic acids have broad-spectrum antibacterial activity and high food safety (GRAS) and are widely used in the food industry as antimicrobial agents [13]. In addition, organic acids have little impact on the sensory properties of products and are a low-cost, easy-to-apply option in the food industry [14], where they can be used in animal production, as growth promoters, and in the food industry for the hygiene of equipment directly related to food [15]. The molecular mechanisms of organic acid inhibition include energy competition, bacterial outer membrane permeabilization, increased intracellular osmotic pressure, and the inhibition of biomolecule synthesis. Undissociated organic acid molecules are lipid-soluble and can enter the cell by free diffusion and dissociate to produce acid ions (ROO^−^) and protons (H^+^). The accumulation of H^+^ in the cytosol leads to cytoplasmic acidification, so the cell releases H^+^ by active transport to maintain the intracellular pH, which consumes large amounts of ATP [16,17]. In the process of releasing H^+^, the cells also exchange and pump in potassium ions, resulting in the destruction of bacterial transmembrane proton motility and further increasing the intracellular osmotic pressure, causing the rupture of the bacterial cytoplasmic membrane and the leakage of contents [18]. The intracellular accumulation of acid ions of organic acids leads to an increase in intracellular osmotic pressure; to balance the intra- and extracellular osmotic pressure, certain precursors and cofactors necessary for bacterial growth are released into the extracellular space, thus inhibiting normal growth and metabolism [19]. Acid ion enrichment in the cell can also interfere with and block DNA synthesis in the nucleus [20], affecting the metabolic transcription of bacterial energy-producing processes [21] and blunting or causing the denaturation of key intracellular enzymes [22].

The commonly used organic acids include acetic acid (AA), lactic acid (LA), butyric acid (BA), citric acid (CA), and malic acid (MA). AA is a dibasic acid with a high dissociative capacity and has the strongest inhibitory effect on Gram-negative bacteria at the same concentration compared to other organic acids, and the inhibitory activity increases with increasing concentration as well as decreasing pH. LA is the most widely used GRAS organic acid and can be used for the disinfection treatment of fresh leafy vegetables [23]. Studies on the inactivation kinetics of *E. coli* have shown that LA is more effective than AA and has no irritating odor [15]. BA is commonly used in poultry feed as a substitute for antibiotics in livestock production systems and exhibits better bacteriostatic effects than acetic acid, formic acid, and propionic acid against certain pathogens [24]. CA and MA, the main organic acids present in fruit, have a high molecular weight, are more easily permeable through cell membranes, and inhibit the growth of pathogenic bacteria mainly by reducing pH and metal-chelating ability [25]. Related studies have shown that when the ambient pH is between pKa1 and pKa2, CA shows similar activity to many membrane-targeted drugs (e.g., EDTA) and kills metabolically inactive cells inside the microcolony, while not affecting the peripheral active cells [26]. Single organic acids need to be used at a certain concentration, which usually leads to higher costs, affects the flavor of the product, and causes the loss of nutrients. Therefore, organic acids can be combined through the synergistic activity of target bacteria to enhance bacterial inhibition and reduce the use of organic acids. Several studies have attempted to combine organic acids for the cleaning and disinfection of meat and fresh vegetables [23,27], which were able to achieve more effective bacterial inhibition. Our previous study showed that the combination of AA and LA has an additive effect, while the combination of AA, LA, and a third organic acid (BA, CA, MA) has a synergistic effect on inhibiting the growth of *E. coli*. However, there are few types of research on the mechanisms underlying the synergistic antibacterial effects of organic acid combinations. Given these concerns, we have preliminarily explored the synergistic inhibition mechanism of organic acid combination by studying their effects on the cell wall, intra- and extracellular membranes, and intracellular proteins of *E. coli*. In addition, the effect of organic acids on the biofilm of *E. coli* was also investigated, including the inhibition rate of biofilm formation, the elimination rate of mature biofilm, and the inhibition rate of cell activity in the biofilm.

## 2. Materials and Methods

### 2.1. Bacterial Strains and Organic Acids

*Escherichia coli* ATCC25922 is conserved by the Key Laboratory of Dairy Biotechnology and Safety Control, Yangzhou University (Yangzhou, China). Before use, *E. coli* cultures were stored in a 1:1 ratio with 70% (*v*/*v*) glycerol solution in a −40 °C refrigerator. *E. coli* was cultured in Luria–Bertani (LB) broth containing 0.5% yeast extract (Macklin Biochemical Co., Ltd., Shanghai, China), 1.0% tryptone (Sinopharm Chemical Reagent Co., Ltd., Shanghai, China), and 1.0% NaCl (Sinopharm Chemical Reagent Co., Ltd., Shanghai, China). The organic acids (AA, LA, BA, CA, and MA) were purchased from Macklin.

Configuration of organic acid solutions: Organic acid solutions of different concentrations for treating *E. coli* are shown in Table 1. Based on our previous research [28], the minimum inhibitory concentration (MIC) was obtained by the microdilution method, and the optimal inhibitory concentration was obtained by the checkerboard method.

### 2.2. Methodology

#### 2.2.1. Alkaline Phosphatase (AKP) Activity Determination

*E. coli* culture at the logarithmic growth stage was centrifuged at 4 °C and 4500 rpm for 10 min, washed three times, 0.85% sterile saline was added to make the bacterial suspension, and the OD_600_ of the strain was adjusted to 0.4. The standard solution of organic acids was added and adjusted to the MIC of each organic acid and the optimal inhibition concentration of the organic acid combination according to Table 1, and saline was used as the control group. The sample was incubated at 37 °C for 4 h followed by centrifugation at 4 °C and 4500 rpm for 10 min. The supernatant was extracted, and the AKP activity was determined using an AKP activity assay kit (JianCheng Bioengineering Institute, Nanjing, China).

#### 2.2.2. Extracellular Nucleic Acids and Proteins Measurement

The leakage of nucleic acids and protein-like substances in *E. coli* cells was detected by the UV absorption method. Refer to Section 2.2.1 for methods of treating *E. coli* with organic acids. Saline was used as the control group. In the study, 37 °C incubation was performed at 0 h, 2 h, 4 h, 6 h, and 8 h. The absorbances were determined by centrifuging the supernatant at 260 nm and 280 nm, which are the characteristic wavelengths for nucleic acids and proteins, respectively. The absorbance values represent the leakage of nucleic acids and proteins from the treated bacteria.

#### 2.2.3. Intracellular Proteins Measurement

The effect of organic acids on the intracellular proteins of *E. coli* was investigated by sodium dodecyl sulfate-polyacrylamide gel electrophoresis (SDS-PAGE). Refer to Section 2.2.1 for methods of treating *E. coli* with organic acids. Saline was used as the control group. After 4 h of incubation, the organisms were washed and concentrated using PBS buffer. An ultrasonic cell crusher VCX800 (SONICS, Newtown, CT, USA) was used to crush the bacterial cells with the following ultrasonic crushing conditions, 2.5 kHz, 200 W, 10 cycles, 10 s pulse, and 30 s stop. Then, the supernatant was collected, and the protein concentration was determined. The buffer (10 μL containing 250 mmol/L Tris–HCl pH 6.8, 10% SDS, 0.5% bromophenol blue, 50% glycerine, and 5% β-mercaptoethanol) was added into 10 μL of samples with a protein concentration of approximately 0.4 mg/mL. The mixture was boiled for 8 min and cooled on ice before SDS-PAGE analysis (the sample volume was 20 μL, and the concentration of the separation gel was 12.5%). The gel was stained with Coomassie brilliant blue R-250 after electrophoresis and then decolorized to obtain the separated protein bands. They were photographed, and data were processed using a Gel Imager GenoSens2000 (Qinxiang Company, Shanghai, China).

#### 2.2.4. OmpF, OmpW, OmpX, OmpA, FadR, and PagP Gene Expression Analysis

Refer to Section 2.2.1 for methods of treating *E. coli* with organic acids. Saline was used as the control group. Total RNA was extracted from *E. coli* using a Trizol Total RNA Extraction Kit UNI1-10 (Novozymes, Nanjing, China) according to the manufacturer’s instructions, and the concentration of the RNA samples was determined using a Nanophotometer N60 (Implen, Munich, Germany). First-strand cDNA was synthesized using a reverse transcription kit, HiScript II Q RT SuperMix for qPCR +wiper (Vazyme, Nanjing China), according to the manufacturer’s instructions. mRNA levels of genes were measured using a real-time fluorescent quantitative PCR system (StepOne plus, Thermo Fisher Scientific, CA, USA). Real-time fluorescence quantitative PCR was established using AceQ Universal SYBR qPCR Master Mix premix (Vazyme, Nanjing China), and 16S rRNA was used as an internal reference gene (Table 2). The expression of target genes in comparison to the control gene 16S rRNA was evaluated using the 2ΔΔCt method, where ΔΔCt is calculated by using Equation (1).

(1) $${\Delta \Delta \mathrm{Ct}=(\mathrm{Ct}}_{\mathrm{target}\;\mathrm{genes}}{\;-\;\mathrm{Ct}}_{16S\;\mathrm{rRNA}}{)}_{\mathrm{treatment}}{\;-\;(\mathrm{Ct}}_{\mathrm{target}\;\mathrm{genes}}{\;-\;\mathrm{Ct}}_{16S\;\mathrm{rRNA}}{)}_{\mathrm{control}}$$

#### 2.2.5. Biofilm Formation, Mature Biofilms, and Cell Activity within Biofilm Measurement

The effect of organic acids on the biofilm formation of *E. coli*; We took a sterile 96-well polystyrene microplate and disinfected it with ultraviolet light. Each well was filled with 100 μL suspension and 100 μL organic acid standard solution and adjusted to the MIC of each organic acid and the optimal inhibition concentration of the organic acid combination according to Table 1. Saline was used as the control group. After incubation at 37 °C for 24 h, the plates were washed three times with 250 μL of distilled water, and the pathogenic cells attached to the wall of the wells were fixed with 250 μL of methanol for 15 min. After emptying the wells and air-drying, 250 μL of 0.1% crystal violet solution was added for 15 min, and the excess crystal violet was removed under running water and air-dried again for 2 h. The crystal violet dye colored in the bacterium was redissolved with 250 μL of 33% (*v*/*v*) acetic acid, and the absorbance was measured at 590 nm.

The inhibition rate of biofilm formation was calculated by the following formula:

(2) $$\mathrm{Inhibition}\;\mathrm{rate}\;\mathrm{of}\;\mathrm{biofilm}\;\mathrm{formation}\;(\%)=\frac{{(\mathrm{OD}}_{590}^{\mathrm{control}}\;{-\;\mathrm{OD}}_{590}^{\mathrm{experiment}})}{{\mathrm{OD}}_{590}^{\mathrm{control}}}\;\text{×}\;100\%$$

The effect of organic acids on the mature biofilm of *E. coli*: A 96-well plate was incubated with a suspension of pathogenic bacteria in a constant temperature incubator at 37 °C for 24 h. After biofilm maturation, the unadhered bacteria were removed and 200 μL organic acid standard solution was added and adjusted to the MIC of each organic acid and the optimal inhibition concentration of the organic acid combination according to Table 1. Saline without organic acids was used as the control. After incubation at 37 °C for 4 h, the relative content of the remaining biofilm in the 96-well plates was determined using crystal violet.

The elimination rate of mature biofilm was calculated by the following formula:

(3) $$\mathrm{Elimination}\;\mathrm{rate}\;\mathrm{of}\;\mathrm{mature}\;\mathrm{biofilm}\;(\%)=\frac{{(\mathrm{OD}}_{590}^{\mathrm{control}}\;{-\;\mathrm{OD}}_{590}^{\mathrm{experiment}})}{{\mathrm{OD}}_{590}^{\mathrm{control}}}\;\text{×}\;100\%$$

The effect of organic acids on the cellular activity of *E. coli* biofilm: The pathogenic bacteria suspension was added to the 96-well plate and incubated in a constant temperature incubator at 37 °C for 24 h. After biofilm maturation, the unadhered bacteria were removed, 200 μL of organic acid standard solution was added and adjusted to the MIC of each organic acid and the optimal inhibition concentration of the organic acid combination according to Table 1. Saline without organic acids was used as the control. The plate was incubated at 37 °C for 4 h. After removing the 96-well plate and aspirating 100 μL of saline from the wells, 10 μL of CCK-8 reagent (Tong Ren Chemical, Kumamoto, Japan) was added, and the plate was incubated at 37 °C and protected from light for 2 h. The absorbance was measured at 450 nm.

The inhibition rate of cell activity in the biofilm was calculated by the following formula:

(4) $$\mathrm{Inhibition}\;\mathrm{rate}\;\mathrm{of}\;\mathrm{cell}\;\mathrm{activity}\;\mathrm{in}\;\mathrm{the}\;\mathrm{biofilm}\;(\%)=\frac{{(\mathrm{OD}}_{450}^{\mathrm{control}}\;{-\;\mathrm{OD}}_{450}^{\mathrm{experiment}})}{{\mathrm{OD}}_{450}^{\mathrm{control}}}\;\text{×}\;100\%$$

### 2.3. Statistical Analysis

All experiments were carried out in triplicate. The experimental data were plotted using Origin 2022 (Origin Lab, Northampton, MA, USA), and SPSS (version 25.0, IBM, Armonk, NY, USA) was used for data analysis.

## 3. Results and Discussion

### 3.1. The AKP Leakage of E. coli

**Figure 1:**
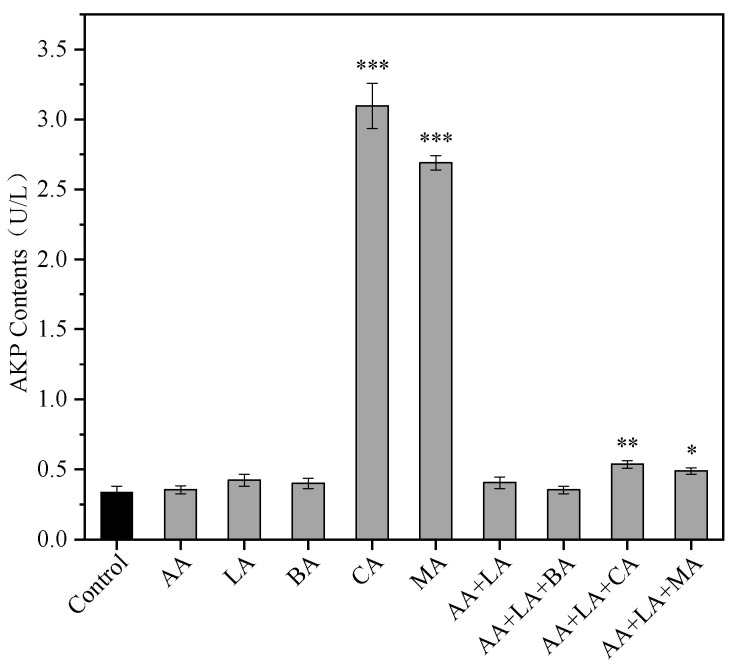
Effect of organic acids and their combinations on the extracellular AKP activity of *E. coli*. The results are expressed as the mean of three experiments (n = 3); error lines indicate standard deviations; * *p* ≤ 0.05, ** *p* ≤ 0.01, and *** *p* ≤ 0.001 versus the control group using the LSD test. AA, acetic acid. LA, lactic acid. BA, butyric acid. CA, citric acid. MA, malic acid (the same applies to the following).

The bacterial cell wall is an important structure that maintains the cell’s osmotic pressure, shape, and integrity and is essential for bacterial viability. The periplasmic space is the narrow space between the outer membrane and the cell wall of Gram-negative bacteria and is susceptible to acid-induced damage [31]. AKP in bacteria is mainly found in the periplasmic space, so extracellular AKP activity can be used to assess the integrity of the bacterial cell wall [32]. The extracellular AKP activity of *E. coli* treated with 1 MIC of CA and MA reached 3.10 U/L and 2.69 U/L, respectively, as shown in Figure 1, which increased 8.16 and 6.95 times, respectively, compared to the control group. However other organic acids had no significant effect on the extracellular AKP activity of *E. coli* (*p* > 0.05). The extracellular AKP activity of AA + LA + CA supplemented with CA and AA + LA + CA supplemented with MA increased by 0.59 and 0.45 times, respectively. The results indicated that 1 MIC of CA and MA could exert bacterial inhibition by disrupting the *E. coli* cell wall, while the combination of organic acids with an optimal inhibition concentration had less effect on the *E. coli* cell wall, which might be related to the low total concentration of organic acids.

Higher concentrations of CA and MA can damage the cell wall of *E. coli*, leading to increased permeability, while other organic acids and the combination at their optimal inhibitory concentration do not cause significant damage to the cell wall of *E. coli*. This may be due to the specific effects of CA and MA on *E. coli* or the lower pH of the medium when these two organic acids reach their MIC of 3.12 and 3.13, respectively, which causes significant damage to the periplasmic space between the cell’s outer membrane and the wall of *E. coli*, leading to increased permeability and AKP leakage.

### 3.2. Nucleic Acid and Protein Leakage of E. coli

**Figure 2:**
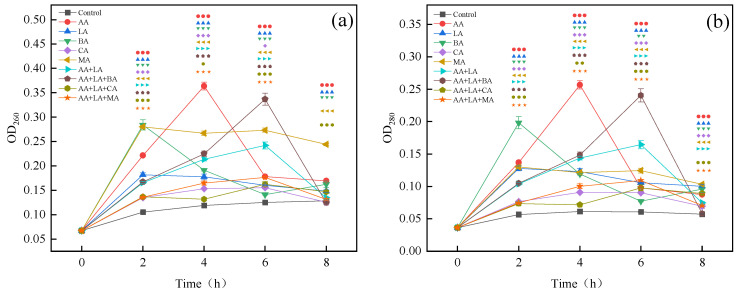
Effect of organic acids and their combinations on the leakage of nucleic acid (**a**) and protein (**b**) from *E. coli*. The results are expressed as the mean of three experiments (n = 3); error lines indicate standard deviations; one symbol: *p* ≤ 0.05, two symbols: *p* ≤ 0.01, three symbols: *p* ≤ 0.001 versus the control group using the LSD test; different symbols represent the different experimental groups as shown in the figures.

Protein and nucleic acid are macromolecules that exist in bacterial cell membranes and cells. They participate in various functions including DNA replication, transcription, and translation. The release of nucleic acid and protein substances reveals the destruction of the integrity of *E. coli* cell membranes by organic acids [33]. Organic acids have the same effect on the leakage of proteins and nucleic acid substances, as shown in Figure 2. According to the highest leakage amount within 0–8 h, the degree of organic acid damage to the *E. coli* cell membranes was ranked high to low as follows, AA > BA > MA > LA > CA. As can be seen from Figure 2, the LA, BA, and MA groups reached the highest leakage at 2 h, the AA group reached the highest leakage at 4 h, and the CA, AA + LA, AA + LA + BA, AA + LA + CA, and AA + LA + MA groups reached the highest leakage at 6 h. This result may indicate that the combination of organic acids can damage the cell membrane of *E. coli* for a longer time than the single organic acid.

Related studies have shown that LA can increase the permeability of the cell membrane of Gram-negative bacteria and improve the antibacterial activity of other drugs [34]. CA destabilizes bacterial cell membranes and promotes the membrane translocation of other weak organic acids, thus enhancing the synergistic antibacterial ability between organic acids [35]. The undissociated small molecule organic acids have different degrees of lipid solubility. These unresolved small molecule organic acids can enter the periplasmic space through the outer membrane pore proteins and protonate the carboxyl and phosphate groups of lipopolysaccharides. This weakens the interactions between the outer membrane components, disrupting the outer membrane integrity and leading to the leakage of contents to achieve the antibacterial function [18]. Therefore, different organic acids act synergistically, as they are highly lipid-soluble organic acids that can increase cell membrane permeability, which may contribute to the synergistic inhibition ability between organic acids. The nucleic acid and protein content in the supernatant began to generally decrease after reaching the highest value as shown in Figure 2, which is similar to that observed by Nan He [36]. This may be caused by nucleic acid and protein being consumed by the remaining bacteria over time or by activating the self-healing mechanisms of bacteria.

### 3.3. Intracellular Protein Analysis

Proteins are large molecules involved in life activities closely related to physiological activities such as metabolism and electron transfer. Organic acids can affect bacterial intracellular proteins by destroying cellular proteins or inhibiting their synthesis to exert an antibacterial effect [37]. Organic acids have multiple effects on bacterial proteins, including interaction with membrane proteins to alter the structure of the cytoplasmic membrane and interfere with cellular energy metabolism [38], causing the unfolding of certain proteins such as HdeA and HdeB in the bacterial cytoplasm, which impairs the acid adaptation ability of *E. coli* [31], and destroying proteins that maintain cell morphology, such as chaperonin and peptidoglycan enzymes, in the periplasmic space of *E. coli* [39].

**Figure 3:**
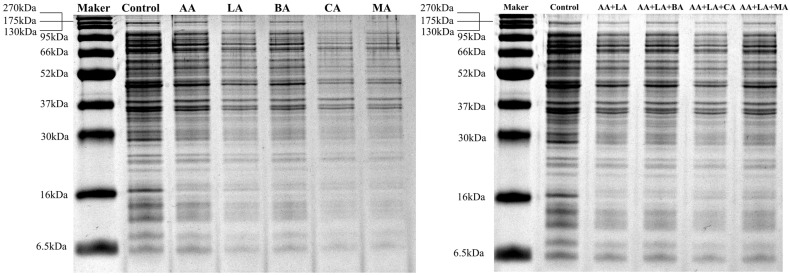
Effect of organic acids and their combinations on the SDS-PAGE intracellular protein profile from *E. coli*.

Compared to the control group, *E. coli* treated with organic acids showed fainter and fewer bands, as seen in Figure 3. As shown in Table 3, 1 MIC of AA, LA, BA, CA, MA, and AA + LA with optimal-inhibitory-concentration-treated *E. coli* showed similarity coefficients of 93.75%, 93.75%, 87.50%, 84.38%, 78.13%, and 93.75%, respectively. Organic acids and their combinations significantly reduced the intracellular protein content of *E. coli*, with the relative protein content of both the CA and MA groups being lower than 50%, 43.99% and 45.11%, respectively. The protein profiles of bacteria can be altered during exposure to a stressed environment. According to the analysis results of the gel imaging system (Table S1), 1 MIC BA, CA, and MA resulted in the disappearance of or extremely weak protein bands, mainly concentrated in the protein bands with molecular weights of 148.67 kDa, 42.59 kDa, and 30.00 ~ 33.82 kDa. These results indicated that these organic acids might exert a bacteriostatic effect by destroying certain proteins with specific functions.

In this study, organic acids and their combinations reduced the relative intracellular protein content of *E. coli*, indicating that organic acids caused the *E. coli* cell membrane damage, which led to the leakage of intracellular proteins, and this part of the study was the same as Figure 2b. The organic acids that strongly affect *E. coli* intracellular proteins, such as CA and MA, may enhance the inhibitory ability of other organic acids by weakening the acid adaptation ability of *E. coli* and destroying some specific functional proteins.

### 3.4. Gene Expression of OmpF, OmpW, OmpX, OmpA, FadR, and PagP

The outer membrane of *E. coli* cells is the main component of the cell wall and consists of outer membrane proteins and phospholipid bilayers, of which outer membrane proteins (OMPs) mainly include lipoproteins and β-barrel transmembrane proteins, which have the role of selectively regulating the entry and exit of substances into and out of cells and are essential for bacterial cell growth as well as providing pathogenicity [40]. In acidic environments, bacterial cell membranes are involved in acid stress response mainly through cell membrane lipids and certain membrane proteins that maintain the integrity and stability of the cell membrane, thereby inhibiting proton influx into the cell interior and further protecting important membrane proteins and pumping proton chains [41].

In this study, the expression of OmpF, OmpW, OmpX, and OmpA in *E. coli* was differentially upregulated after treatment with different organic acids and their combinations, as shown in Table 4. OmpF is mainly involved in the transport of carbohydrates, ions, antibiotics, and proteins across the outer membrane, and usually in acidic environments, the expression of OmpF decreases to increase the acid tolerance of *E. coli* [42]. However, in low-nutrient environments, it is crucial to accelerate the absorption of nutrients, and the increase in OmpF expression helps the growth of *E. coli* under low osmotic pressure [43]. During the experiments in this paper, *E. coli* was in a low-nutrient environment, so the organic acids and their combinations significantly increased the relative expression of OmpF, up to 27.25-fold in the LA group and down to 9.04-fold in the AA + LA + BA group, as shown in Table 4. The results suggest that the increased expression of OmpF in acidic conditions with low osmolarity may contribute to the entry of organic acids into the cell interior, accelerating bacterial cell death.

OmpW, OmpX, and OmpA are small OMPs consisting of eight to ten transmembrane β-barrel structures involved in the specific transport of nutrients, etc. These proteins are highly conserved in most Gram-negative bacteria and have regulatory effects on many functions. OmpW has a role in the transmembrane uptake of small hydrophobic molecules and the regulation of bacterial iron ion homeostasis [44]. OmpX can reduce the susceptibility of bacteria to certain drugs and lead to the development of drug resistance [45]. OmpA helps maintain the structural integrity of the bacterial outer membrane and bacterial cell morphology [46]. In this study, 1 MIC of AA, CA, and MA all significantly (*p* < 0.05) increased the relative expression of OmpW and OmpA with limited effect on the expression of OmpX, while 1 MIC of LA and 1 MIC of BA increased the relative expression of OmpW and OmpX, respectively, with no significant difference in the increase in OmpA expression, as shown in Table 4. All organic acid combinations, AA + LA, AA + LA + BA, AA + LA + CA, and AA + LA + MA, significantly (*p* < 0.05) increased the relative expression of OmpW, and the AA + LA group increased the relative expression of OmpX by 10.87 fold but had less effect on the expression of OmpA. The AA + LA + BA group increased the relative expression of OmpA by 8.14 fold. However, the effect of the AA + LA + BA group on the relative expression of OmpX was not significant (*p* > 0.05) as shown in Table 4. PagP is the only known LPS in the *E. coli* biosynthetic extracellular membrane enzyme with a role in maintaining bacterial outer membrane asymmetry and stability. The changes in its expression were associated with changes in the lipid layer [30,47]. In this study, except MA at 1 MIC, all the organic acids and their combinations increased the expression of PagP to different degrees, and the relative expression of PagP in *E. coli* after AA treatment with 1 MIC was the highest, 21.76.

The increased expression levels of OmpW, OmpX, OmpA, and PagP indicated that *E. coli* was affected by organic acids and thus activated the acid adaptation responses of the extracellular membrane. The relative expression of OmpX, OmpA, and PagP was differently affected by different organic acids, indicating that the defense mechanisms of the *E. coli* outer membrane against different organic acid attacks were different. The expression of OMPs was affected by the type and concentration of organic acids.

Another important membrane structure of *E. coli* is the cytoplasmic membrane (inner membrane) composed of phospholipids and proteins, which are involved in nutrient uptake and metabolite transfer, and the inner membrane is essential for the maintenance of normal bacterial life activities [48]. FadR is a global regulator of the fatty acid pathway and is involved in multiple processes of fatty acid biosynthesis, degradation, and transmembrane transport [49]. In this study, the relative expression of FadR increased more than two fold after organic acids and their combinations were treated with the highest 27.11-fold in the CA group. The upregulation of FadR expression induced by organic acids indicates that *E. coli* attempts to repair the damage to the cytoplasmic membrane by promoting the synthesis of the phospholipid bilayer.

### 3.5. Effects of Organic Acids and Their Combinations on Biofilm Formation, Mature Biofilms, and Cell Activity within Biofilms in E. coli

**Figure 4:**
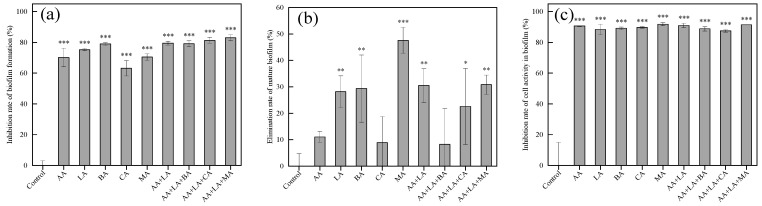
Effects of organic acids and their combinations on biofilm formation (**a**), mature biofilm (**b**), and cell activity in the biofilm (**c**) of *E. coli*. The results are expressed as the mean of three experiments (n = 3); error lines indicate standard deviations; * *p* ≤ 0.05, ** *p* ≤ 0.01, and *** *p* ≤ 0.001 versus the control group using the LSD test.

In the food industry, once foodborne pathogens are attached to food-contact surfaces (such as workbenches, storage tanks, and kitchenware), they are likely to form biofilms as their survival strategy, leading to subsequent food spoilage and even foodborne diseases [50]. In this study, the effects of organic acids on biofilm formation, cell viability within the biofilm, and the mature biofilm of *E. coli* were investigated using crystal violet and the CCK-8 cell viability assay. As shown in Figure 4, the inhibition rate of biofilm formation of organic acids and their combinations ranged from 63.15% to 82.96%, and the inhibition rate of cell activity in the biofilm ranged from 88.32% to 91.82%. The elimination ability of mature biofilm by organic acids and their combinations varied greatly and was less than 50%, ranging from 8.29% to 47.60%.

Available studies have shown that organic acids can inhibit the biofilm formation of foodborne pathogens, which may be related to a reduction in the expression of the signaling molecule AI-2 [51]. B Amrutha et al. [52] showed that AA, CA, and LA all played an inhibitory role in the biofilm formation of *E. coli*. It has also been shown that MA and CA exhibit certain anti-biofilm activity at different temperatures and concentrations [25]. Previous studies have shown that combining fumaric acid, lactic acid, and ferulic acid can effectively combat the biofilm of various foodborne bacteria [53]. The application of a combination of malic acid and lactic acid electrostatic spray can improve the microbial safety of spinach and cantaloupe by preventing pathogenic biofilm formation and bacterial growth [54]. As shown in Figure 4a, the inhibition rate of *E. coli* biofilm formation by combining organic acids with the minimum inhibitory concentration is between 79.17 ~ 82.96%, generally higher than that of a single organic acid at 1 MIC. Therefore, the combination of organic acids may have a synergistic effect in inhibiting *E. coli* biofilm formation.

In the process of biofilm formation, EPS produced by microorganisms will limit the diffusion of disinfectants into the biofilm, making the biofilm play a role in protecting pathogenic bacteria from adverse conditions and antibacterial agents [55]. Biofilms have a complex matrix of EPS that envelopes microorganisms on the surface, making biofilm removal more difficult and requiring the use of intense shear forces (scraping or scrubbing) or the chemical breakdown of the adhesion forces by utilizing antimicrobial substances or heat [56]. Figure 4b shows that the effects of different organic acids on the mature biofilm are quite different. CA at 1 MIC and AA + LA + BA at the optimal inhibitory concentration have the lowest elimination rate of 8.89% and 8.29% on the mature biofilm, respectively, while MA at 1 MIC has the highest elimination rate of 47.60%. Studies have shown that the combined treatment of ultrasound and organic acids can effectively remove *E. coli* biofilm formed on lettuce leaves and has a synergistic effect [57]. Both organic acids and their combinations inhibited the cell activity within the *E. coli* biofilm by about 90%, as shown in Figure 4c. Therefore, the use of organic acid combinations as disinfectants for *E. coli* with established biofilms has significant advantages, and the combination with other physical means can also be effective for the removal of mature biofilms.

## 4. Conclusions

The results obtained in the current study demonstrated that AA, LA, BA, CA, and MA had different effects on the cell barrier and biofilm of *E. coli*. AA was the most destructive to the cell membrane, followed by BA, CA, and MA, which had specific destructive effects on the cell wall and the most destructive effects on the intracellular proteins, leading to the disappearance of some protein bands. The results of RT-qPCR showed that the expressions of the extracellular membrane proteins OmpF, OmpW, OmpX, OmpA, and PagP and the intracellular membrane protein FadR were upregulated to varying degrees by organic acids and their combinations. Moreover, organic acids and their combinations could inhibit the formation of the biofilm and the cell activity in the biofilm by about 90%, but the elimination rate of mature biofilm is lower than 50%. Overall, the combination of organic acids can exert a good antibacterial effect by destroying the cell barrier and may have a synergistic anti-biofilm effect. There may be a variety of external environments and internal conditions in actual application scenarios. Therefore, future research should focus on studying the antibacterial characteristics of organic acid combinations under different use conditions based on the existing theoretical basis, combined with other antibacterial and fresh-preserving technologies (chemical, physical, and biological), and designing improved antibacterial strategies based on effective synergistic activities or additive effects to meet the requirements of modern consumers for food safety and quality.

## Figures and Tables

**Table 1:** MIC of the organic acids and optimal inhibitory concentration of the organic acid combination.

Single Organic Acid ^3^	MIC ^1^ (μg/mL)	Organic Acid Combination ^3^	Optimal Inhibitory Concentration ^2^ (μg/mL)
AA	2560	AA + LA	640 + 640
LA	2560	AA + LA + BA	320 + 320 + 320
BA	2560	AA + LA + CA	80 + 80 + 1280
CA	5120	AA + LA + MA	320 + 320 + 640
MA	5120		

^1^ The MIC of the organic acid represents the lowest drug concentration at which a single organic acid can inhibit the growth and reproduction of microorganisms. ^2^ The optimal inhibitory concentration of the organic acid combination represents the lowest drug concentration at which a combination of organic acids can inhibit the growth and reproduction of microorganisms. ^3^ AA, acetic acid. LA, lactic acid. BA, butyric acid. CA, citric acid. MA, malic Acid.

**Table 2:** Sequences characteristics of oligonucleotide primers used in this study and target genes description.

Target Genes	Sequence (5′-3′)	Description	Reference
16S rRNA	F: AGAGGATGACCAGCCACAC R: CGGGTAACGTCAATGAGCAAAG	Reference gene	[29]
OmpF	F: CGGTTATGGTCAGTGGGA R: CGAAGAAGTCATCGCTGTAT	Outer membrane porin sequencing of CDC ceftolozane/tazobactam antimicrobial resistance panel	
OmpW	F: TGCTGGTGGTACGTTAGGAA R: TGTTGGTGGCAGATGATGAA	Associated with the transport of small molecules	
OmpX	F: AACGCTACGAATACGGCTCT R: TACCCGACCTACAAACACGA	Causes the development of drug resistance	
OmpA	F: TGAGCCTGGGTGTTTCCTAC R: ATCCAGAGCAGCCTGACCTT	Maintains outer membrane integrity, stable cell structure, and cell morphology	
FadR	F: GATAATTTGCTGTCGGTGCG R: CCGGTTCCGACTGGCTGGAA	Transcriptional regulator of fatty acid metabolism	[30]
PagP	F: GCTAACGCAGATGAGTGGATGACAAC R: CACGAGTCCTTAAATGCCATGG	Phospholipid/lipid A palmitoyl transferase	

**Table 3:** Effect of organic acids and their combinations on the intracellular proteins of *E. coli*.

Group	Similarity Coefficient ^1^ (%)	Total Relative Protein Content ^2^(%)
Control	100.00	100.00
AA	93.75	75.12
LA	93.75	50.21
BA	87.50	64.57
CA	84.38	43.99
MA	78.13	45.11
AA + LA	93.75	60.95
AA + LA + BA	100.00	77.17
AA + LA + CA	100.00	57.64
AA + LA + MA	100.00	74.19

^1^ Similarity coefficient (%) = the number of bands with similar molecular weight to the control group/the number of bands in the control group. ^2^ Total relative protein content (%) = total relative protein content of SDS-PAGE in the organic acid group/total relative protein content of SDS-PAGE in the control group; the total relative protein content of SDS-PAGE is calculated as the sum of the optical density value of each protein band in the gel electrophoresis map × molecular weight.

**Table 4:** Effect of organic acids and their combinations on gene expression in *E. coli*.

Group	Relative Gene Expression ^1^					
	OmpF	OmpW	OmpX	OmpA	FadR	PagP
Control	1.02 ± 0.19 ^g^	1.01 ± 0.12 ^f^	1.00 ± 0.03 ^e^	1.01 ± 0.11 ^e^	1.00 ± 0.05 ^f,g^	1.00 ± 0.03 ^g^
AA	12.72 ± 1.29 ^e,f^	10.14 ± 0.46 ^b,c,d^	1.85 ± 0.39 ^d,e^	9.05 ± 1.14 ^a^	14.76 ± 0.25 ^b^	21.76 ± 0.40 ^a^
LA	27.25 ± 1.20 ^a^	6.94 ± 0.49 ^d,e^	5.45 ± 0.57 ^c^	2.15 ± 0.12 ^d,e^	7.62 ± 0.87 ^c,d^	1.85 ± 0.11 ^f^
BA	15.86 ± 0.52 ^c,d,e^	18.50 ± 3.00 ^a^	7.52 ± 0.69 ^b^	2.08 ± 0.40 ^d,e^	3.94 ± 0.62 ^e,f,g^	2.26 ± 0.15 ^e,f^
CA	21.95 ± 5.37 ^a,b,c^	9.11 ± 0.56 ^b,c,d^	1.63 ± 0.06 ^d,e^	7.18 ± 0.21 ^b^	27.11 ± 3.16 ^a^	2.44 ± 0.07 ^e^
MA	23.80 ± 2.66 ^b,c^	5.19 ± 1.91 ^e^	1.93 ± 0.31 ^d,e^	3.96 ± 0.50 ^c^	8.45 ± 0.77 ^c^	1.05 ± 0.14 ^g^
AA + LA	13.58 ± 1.20 ^d,e,f^	11.10 ± 1.34 ^b,c^	10.87 ± 0.81 ^a^	2.23 ± 0.08 ^d,e^	4.91 ± 0.76 ^d,e,f^	2.98 ± 0.10 ^d^
AA + LA + BA	9.04 ± 1.76 ^f^	12.48 ± 0.88 ^b^	1.55 ± 0.08 ^d,e^	2.98 ± 0.46 ^c,d^	6.33 ± 0.64 ^c,d,e^	3.96 ± 0.04 ^c^
AA + LA + CA	18.99 ± 2.67 ^b,c,d,e^	12.49 ± 0.97 ^b^	2.02 ± 0.22 ^d,e^	8.14 ± 0.72 ^a,b^	16.61 ± 1.21 ^b^	10.29 ± 0.17 ^b^
AA + LA + MA	19.55 ± 2.65 ^b,c,d^	7.75 ± 0.19 ^c,d,e^	2.32 ± 0.61 ^d^	3.66 ± 0.27 ^c^	2.84 ± 0.33 ^f,g^	1.81 ± 0.05 ^f^

^1^ The results are expressed as the mean ± standard deviation, and different lowercase letters indicate statistical differences between the different organic acid groups (*p* < 0.05).

## Data Availability

Date is contained within the article and Supplementary Material.

## Supplementary Materials

The following supporting information can be downloaded at: https://www.mdpi.com/article/10.3390/foods12163011/s1. Table S1: The analysis results of SDS-PAGE.
